# Vaginal microbiota of adolescents and their mothers: A preliminary study of vertical transmission and persistence

**DOI:** 10.3389/frmbi.2023.1129394

**Published:** 2023-03-24

**Authors:** Christine M. Bassis, Kaylie A. Bullock, Daniel E. Sack, Katie Saund, Ali Pirani, Evan S. Snitkin, Veronica I. Alaniz, Elisabeth H. Quint, Jason D. Bell, Vincent B. Young

**Affiliations:** ^1^ Department of Internal Medicine/Division of Infectious Diseases, University of Michigan Medical School, Ann Arbor, MI, United States; ^2^ Department of Microbiology & Immunology, University of Michigan Medical School, Ann Arbor, MI, United States; ^3^ Department of Obstetrics and Gynecology, University of Michigan Medical School, Ann Arbor, MI, United States

**Keywords:** vaginal microbiota, vertical transmission, microbiome, 16S rRNA gene sequences, Lactobacillus crispatus genomics, mother-daughter vaginal microbiota

## Abstract

**Introduction:**

Factors that influence vaginal microbiota composition, including its source, are not well understood.

**Methods:**

To determine if vaginal microbiota transmission from mother to daughter at birth could possibly influence the human vaginal microbiota composition in adolescence, we conducted a preliminary study to investigate the relationship between the vaginal microbiota of 13 adolescents (ages 15-21) and their mothers and the daughter’s birth mode.

**Results and discussion:**

Based on analysis of bacterial 16S rRNA gene sequences, the vaginal microbiotas of mother/daughter pairs were more similar to each other if the daughter was born by vaginal delivery rather than by C-section. Specifically, within pair β-diversity, measured by the Yue and Clayton θ (θYC) distance metric, was significantly lower if the daughter was born by vaginal delivery. Additionally, genome sequences from an important member of the vaginal microbiota, *Lactobacillus crispatus*, isolated from one mother/daughter pair in which the daughter was born by vaginal delivery, were highly similar based on recombination-filtered single nucleotide polymorphisms (SNPs). Both community-level analysis and isolate genome sequence analysis are consistent with birth-mode dependent transmission and persistence of at least some members of the vaginal microbiota.

## 1 Introduction

The vaginal microbiota plays an important role in human health. The community structure of the vaginal microbiota is linked to infection susceptibility and preterm birth (Callahan et al., 2017; Klatt et al., 2017; McClelland et al., 2018; Bayigga et al., 2019; Tamarelle et al., 2019; Sun et al., 2022). The composition of the vaginal microbiota is distinct from other body sites and contains types of bacteria that seem specific to this anatomic location (Human Microbiome Project Consortium, H.M.P, 2012). For example, the vaginal microbiota is often dominated by specific types of *Lactobacillus*, most commonly *L. crispatus* and *L. iners* (Ravel et al., 2011; Bassis et al., 2017). Vaginal *Lactobacillus* sp. are thought to maintain dominance and inhibit colonization of other microbes through lactic acid production (O’Hanlon et al., 2013; Tachedjian et al., 2018).

Despite strong evidence that the vaginal microbiota can have significant impacts on health, the factors that influence the composition of the vaginal microbiota are not well understood. It is not known how this vagina-specific community is maintained from generation to generation. One possibility is that at least some members of the vaginal microbiota are transmitted from mother to daughter at birth and maintained in daughters through adolescence.

In healthy babies, the first large, direct exposure to microbes occurs at birth. Birth mode has been shown to influence the composition of the newborn microbiota (gut, skin, mouth), likely due to different bacterial exposure in vaginal delivery and C-sections (Dominguez-Bello et al., 2010; Madan et al., 2016). However, the effect of birth mode on the composition of the vaginal microbiota has not been investigated. In this study, we compared the vaginal microbiotas of 13 mother/daughter pairs and investigated the effect of birth mode on mother/daughter microbiota similarity. We also compared the genome sequences from *Lactobacillus crispatus* isolates from one mother/daughter pair. In this study, we took the first steps toward addressing our hypothesis that the vaginal microbiota of mothers and daughters would be more similar if the daughter was born by vaginal delivery than by C-section.

## 2 Materials and methods

### 2.1 Subject recruitment and sample collection

Mother/daughter pairs were recruited from the Pediatric and Adolescent Gynecology Clinic at the University of Michigan Health System in 2014 and 2015. Exclusions were pregnancy and age of less than 15 years. Written, informed consent was obtained and participants completed a baseline Qualtrics survey on their demographics and pertinent gynecologic and medical history. Vaginal samples were self-collected using a dual-headed swab (Starplex Scientific, S09D) at baseline and then weekly for 4 weeks. The baseline swab was obtained in the clinic, with immediate storage on ice and transfer to -80°C within a few hours. The subsequent swabs were returned *via* mail at ambient temperature. For each swab, subjects were asked to record the sampling date and if they were menstruating at the time of sampling. After the fifth swab was received and a completion incentive was mailed to the subject, the link between samples and subject names was destroyed, irreversibly de-identifying all samples. The study was approved by the University of Michigan IRB (HUM00086661).

### 2.2 DNA isolation and 16S rRNA gene sequencing

One of the swab heads from each sample was clipped directly into the bead plate of a PowerMag Microbiome RNA/DNA Isolation Kit (Mo Bio Laboratories, Inc.). DNA isolation was performed according to the manufacturer’s instructions using an epMotion 5075 liquid handling system. The V4 region of the 16S rRNA gene was amplified from 1 or 7μl DNA and sequenced with a MiSeq (Illumina, San Diego, CA) using the 500-cycle MiSeq Reagent Kit, v. 2 (Illumina, catalog No. MS-102–2003) by the University of Michigan Microbial Systems Molecular Biology Laboratory as described previously (Seekatz et al., 2015). The other swab head was used for cultivation or stored at -80°C.

### 2.3 Bacterial community analysis

The 16S rRNA gene sequences were processed using mothur v.1.36.1 and v.1.39.5 following the mothur MiSeq SOP (Schloss et al., 2009; Kozich et al., 2013). Details of the processing steps are available in mother.daughter_mothur.batch (https://github.com/cbassis/MotherDaughter_Vaginal_Microbiota.study). After sequence processing and alignment to the SILVA reference alignment (Release 102) (Schloss, 2009), sequences were binned into operational taxonomic units (OTUs) based on 97% sequence similarity using the average neighbor method (Schloss and Westcott, 2011; Westcott and Schloss, 2015). Samples with fewer than 1000 sequences were excluded from the analysis. OTUs were classified to the genus level within mothur using a modified version of the Ribosomal Database Project (RDP) training set (version 9) (Wang et al., 2007; Cole et al., 2014). To further classify the *Lactobacillus* OTUs, representative sequences were analyzed using standard nucleotide BLAST for highly similar sequences (megablast) on the National Center for Biotechnology Information (NCBI) BLAST web page (https://blast.ncbi.nlm.nih.gov/Blast.cgi) (Morgulis et al., 2008). OTU relative abundances were calculated and plotted in a heatmap. To compare bacterial communities between pairs, within pairs and within subjects, we calculated θ_YC_ distances (a metric that takes relative abundances of both shared and non-shared OTUs into account) (Yue and Clayton, 2005). A Kruskal-Wallis test with a Dunn’s posttest or a Wilcoxon (Mann-Whitney) test were used to determine if differences in θ_YC_ distances were statistically significant. Principal coordinates analysis (PCoA) was used to visualize the θ_YC_ distances between samples. R Studio (Version 1.1.456) with R (Version 3.5.1) was used for the statistical tests and plotting the heat map, box and whisker plots, and the ordination using the code available: https://github.com/cbassis/MotherDaughter_Vaginal_Microbiota.study/tree/master/R_code. Adobe Illustrator (CS6) was used for labeling and formatting figures.

### 2.4 *Lactobacillus crispatus* isolation

For pair I, the second swab head from the freshly collected baseline vaginal sample was swabbed onto an MRS agar plate and incubated in an anaerobic chamber (Coy Laboratory Products) at 37°C. DNA from individual isolates was amplified with primers targeting the V1-V5 region of the 16S rRNA gene (“B” adapter oligo sequence +27F (5’-CCTATCCCCTGTGTGCCTTGGCAGTCTCAGAGAGTTTGATCCTGGCTCAG-3’) and XLR_926R_v2bBar8L (5’-CCATCTCATCCCTGCGTGTCTCCGACTCAGCACGCCCGTCAATTCMTTTRAGT-3’)) (Jumpstart Consortium Human Microbiome Project Data Generation Working Group, 2010) under the following PCR conditions: 2 minutes @ 95°C; 30x(20 seconds @ 95°C, 30 seconds @ 50°C, 30 sseconds @ 72°C); 5 minutes @ 72°C. Amplicon sequences obtained by *via* Sanger sequencing were used for isolate identification.

### 2.5 DNA isolation and genome sequencing

Three *Lactobacillus crispatus* isolates from pair I, 2 from the mother and 1 from the daughter, were grown overnight in 1 ml liquid MRS in an anaerobic chamber (Coy Laboratory Products) at 37°C. Genomic DNA was isolated from the liquid cultures using the PowerMicrobiome™ RNA Isolation Kit (Mo Bio Laboratories, Inc.) without the DNase treatment. Genome sequencing was performed by the Microbial Systems Molecular Biology Laboratory at the University of Michigan using a Nextera™ XT DNA Library Preparation kit (Illumina, San Diego, CA) and a 600-cycle MiSeq Reagent Kit, v. 3 (Illumina, San Diego, CA) on a MiSeq (Illumina, San Diego, CA).

### 2.6 Genome sequence analysis

Phylogenetic relationships between *L. crispatus* isolates from mother/daughter pair I and all *L. crispatus* strains with genome sequences available as fastq files from NCBI on December 27th, 2018 were determined based on recombination-filtered single nucleotide polymorphisms (SNPs). Quality of reads was assessed with FastQC v0.11.3 (Andrews), and Trimmomatic 0.36 (Bolger et al., 2014) was used for trimming adapter sequences and low-quality bases. Variants were identified by (i) mapping filtered reads to reference genome sequence *L. crispatus* ST1 (SAMEA2272191) using the Burrows-Wheeler short-read aligner (bwa-0.7.17) (Li and Durbin, 2009), (ii) discarding polymerase chain reaction duplicates with Picard (picard-tools-2.5.0), and (iii) calling variants with SAMtools (samtools-1.2) and bcftools. Variants were filtered from raw results using GATK ‘s (GenomeAnalysisTK-3.3-0) VariantFiltration (QUAL, >100; MQ, >50; >=10 reads supporting variant; and FQ, <0.025) (McKenna et al., 2010). In addition, a custom python script was used to filter out single-nucleotide variants that were (i) <5 base pairs (bp) in proximity to indels, (ii) fell under Phage and Repeat region of the reference genome (identified using Phaster (Pon et al., 2016) and Nucmer (MUMmer3.23) (Kurtz et al., 2004)), (iii) not present in the core genome, or (iv) in a recombinant region identified by Gubbins 2.3.1 (Croucher et al., 2015). A maximum likelihood tree was constructed in RAxML 8.2.8 (Stamatakis, 2014) using a general-time reversible model of sequence evolution. Bootstrap analysis was performed with the number of bootstrap replicates determined using the bootstrap convergence test and the autoMRE convergence criteria (-N autoMRE). Bootstrap support values were overlaid on the best scoring tree identified during rapid bootstrap analysis (-f a). The final maximum likelihood tree was plotted and pairwise SNP distances were calculated in R Studio (Version 1.1.463) with R (Version 3.5.3): https://github.com/cbassis/MotherDaughter_Vaginal_Microbiota.study/blob/master/R_code/Mother_Daughter_Figure_3_Genome_Tree_and_Genome_Analysis.Rmd. Adobe Illustrator (CS6) was used for labeling and formatting the figure.

### 2.7 Calculation of doubling time estimate for vaginal *L. crispatus in vivo*

We used the number of SNPs between the pair I mother and daughter *L. crispatus* isolates to estimate the doubling time of vaginal *L. crispatus in vivo* if all SNPs in the recombination-filtered core genome were due to mutations acquired since the daughter’s birth:


$$Doubling\;time=\;\frac{\left(mutation\;rate\right)\times \left(daughters\;age\right)\times \left(genome\;length\right)}{\left(\#\;of\;mutations\right)\;}$$

The mutation rate of *L. crispatus* is unknown, so for this estimate we used the published mutation rate of another *Lactobacillus, L. casei* Zhang, *in vitro*, without antibiotics (1.0x10^-9^ bp/generation) (Wang et al., 2017). The pair I daughter’s age in hours was:


175,200 hours=(20 years)×(365 days per year)×(24 hours per day)

The average length of the recombination-filtered core genome (940,943 bp) was used for genome length. We assumed that the isolates arose from a common ancestor and that all mutations were non-convergent, so the number of mutations acquired by each isolate would equal the number of SNPs between the mother’s isolate and the daughter’s isolate divided by 2. We also estimated the number of mutations acquired per isolate core genome per year as:


$$\frac{\#\;of\;mutations}{daugter^{\prime }s\;age}=\frac{\#\;of\;SNPs}{2\left(daugter^{\prime }s\;age\right)}$$

## 3 Results

### 3.1 Subject characteristics and sequencing results

A total of 107 self-collected, vaginal swab samples were obtained from 26 subjects (13 mother/daughter pairs) (**Table 1** and **Supplemental Table 1**). Each subject returned 1-5 weekly samples (median=5 samples/subject, IQR=1). After sequence processing and exclusion of samples with fewer than 1000 sequences, a total of 2,336,437 high quality bacterial 16S rRNA gene sequences from 101 samples were analyzed with an average of 23,133 +/- 10,212 sequences per sample. Sequence counts and α-diversity metrics for each sample included in our analysis are listed in **Supplemental Table 2**.

**Table 1:** Subject Characteristics.

	Mother (n=13)		Daughter (n=13)	
	Daughter’s birth mode: Vaginal (n=10)	Daughter’s birth mode: C-section (n=3)	Daughter’s birth mode: Vaginal (n=10)	Daughter’s birth mode: C-section (n=3)
**Age, mean ± SD, years**	44.8 ± 5.6	54 ± 2.4	17.1 ± 2.0	18.7 ± 1.9
**Race: White (vs. Black, Asian, Hispanic, other)**	90% (n=9)	100% (n=3)	90% (n=9)	100% (n=3)
**Subject Birth mode: Vaginal (vs. C-section)**	70% (n=7)	100% (n=3)	100% (n=10)	0% (n=0)
**Reproductive stage: Premenarchal**	0% (n=0)	0% (n=0)	10% (n=1)	0% (n=0)
**Reproductive stage: Reproductive**	70% (n=7)	33% (n=1)	90% (n=9)	100% (n=3)
**Reproductive stage: Postmenopausal**	30% (n=3)	67% (n=2)	0% (n=0)	0% (n=0)

### 3.2 An individual’s vaginal microbiota is relatively stable over 4 weeks

**Figure 1:**
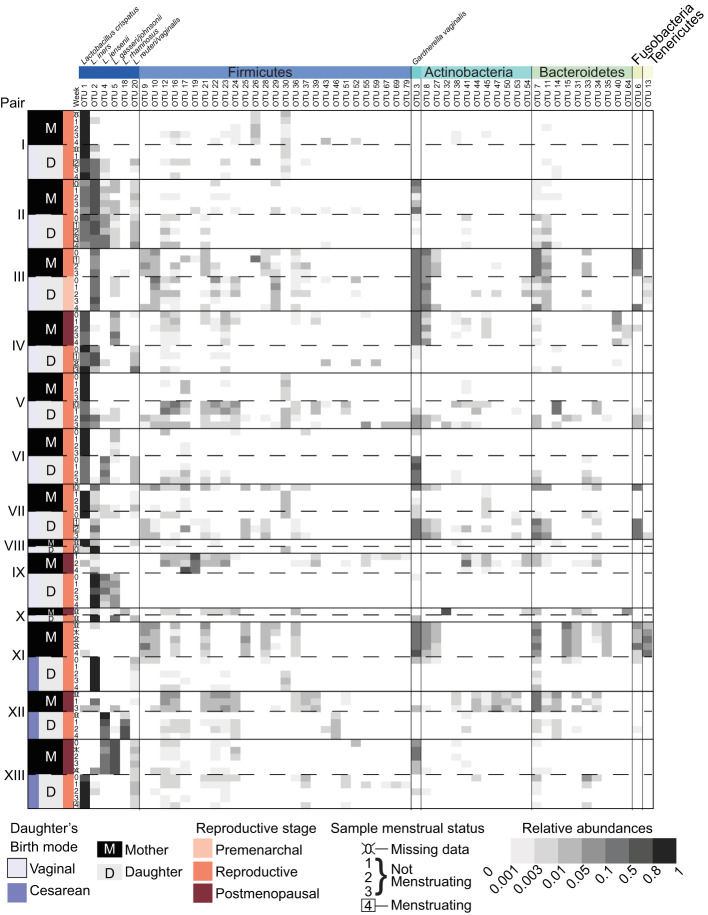
Vaginal bacterial community compositions of mother/daughter pairs. Relative abundances of OTUs in weekly vaginal swab samples from 13 mother/daughter pairs. Mother/daughter pairs were ordered by average within pair θ_YC_ distances, with the most similar pair (I) on top and the least similar pair (XIII) on the bottom. OTUs with a minimum of 200 sequences in the dataset overall and present at a relative abundance greater than 2% in at least 1 sample were included in the heat map. Taxonomic classifications to genus level of these OTUs are listed in **Supplemental Table 3**.

**Figure 2:**
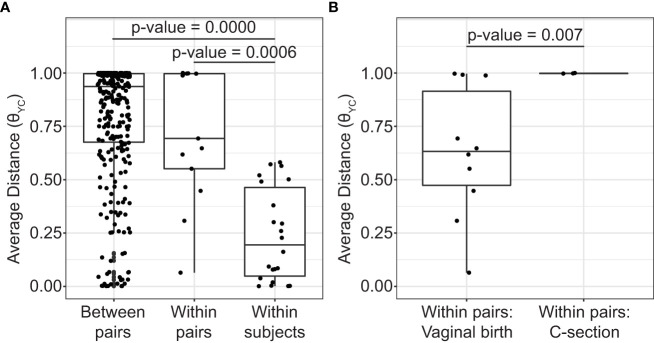
Average distances between vaginal bacterial communities. **(A)** Mean θ_YC_ distances between subjects from different mother/daughter pairs (between pairs), between subjects within a mother/daughter pair (within pair) and between samples from the same subject (within subject). P-values for comparisons that were significantly different by Dunn’s posttest are shown (Kruskal-Wallis p-value= 8.154e-10). **(B)** Mean θ_YC_ distances between subjects within a mother/daughter pair for daughters born by vaginal birth and by C-section. Wilcoxon (Mann-Whitney) test p-value is shown. In the box and whiskers plots, the median θ_YC_ distance is indicated by a line, values within the first to the third quartiles are inside the box and the whiskers extend to the smallest and largest values within 1.5x the interquartile range.

During the sampling period, the vaginal microbiota of each subject was relatively stable. The high stability of the vaginal microbiota is apparent from the consistent within subject community composition (**Figure 1** and **Supplemental Table 3**). For example, OTU1 (*L. crispatus*) and/or OTU2 (*L. iners*) persisted from week to week in many subjects. Additionally, average θ_YC_ distances were significantly lower within subjects than between subjects (Kruskal-Wallis p-value= 8.154e-10) (**Figure 2A**) and samples clustered by subject in a PCoA based on θ_YC_ distances (**Supplemental Figure 1**).

### 3.3 Factors associated with similarity between vaginal microbiotas of mothers and their daughters

To determine if mothers and their daughters had more similar vaginal microbiotas than unrelated subjects, we compared the average θ_YC_ distances between all unrelated subjects (between pairs) and the average θ_YC_ distances between mothers and their own daughters (within pairs) (**Figure 2A**). There was a trend toward greater similarity (lower θ_YC_ distances) within all mother/daughter pairs than between subjects in different mother/daughter pairs. To determine if birth mode was related to vaginal microbiota similarity within mother/daughter pairs, we compared the average within pair θ_YC_ distances for pairs in which the daughter was born by vaginal delivery and by C-section (**Figure 2B**). The average within pair θ_YC_ distances were significantly lower for pairs in which the daughter was born by vaginal delivery compared to C-section (Wilcoxon test p-value=0.007) (**Figure 2B**). Additional factors, including reproductive status, are also likely to contribute to mother/daughter vaginal microbiota similarity. Indeed, we also found a strong trend toward greater mother-daughter vaginal microbiota similarity (lower within pair θ_YC_ distances) if the mother was in her reproductive stage rather than postmenopausal (Wilcoxon test p-value=0.07) (**Supplemental Figure 2**).

### 3.4 *Lactobacillus crispatus* isolates from mother/daughter pair I have highly similar genome sequences

**Figure 3:**
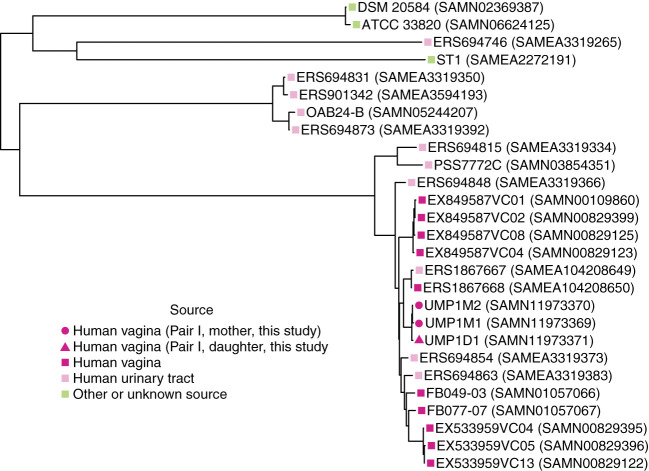
Phylogenetic relationships between *L. crispatus* strains. Maximum likelihood tree based on recombination-filtered SNP distances between *L. crispatus* genome sequences of isolates from mother/daughter pair I and other *L. crispatus* strains with publicly available genomes. Tip labels indicate *L. crispatus* strain names and NCBI BioSample identifiers. Bootstrap values were greater than or equal to 0.65.

The birth mode-dependent similarity of the vaginal microbiotas of mothers and their daughters suggested that vaginal bacteria could be transmitted between generations at birth and persist into adolescence. However, it is possible that genetic or environmental factors shared by a mother and her daughter lead to acquisition of similar bacteria later, resulting in the *de novo* establishment of similar vaginal communities. To investigate the possibility of direct transmission and persistence of one member of the vaginal microbiota, we generated draft genome sequences of *Lactobacillus crispatus* strains isolated from the freshly collected second swab head of mother/daughter pair I. The draft genome sequences of these isolates were compared with publicly available *L. crispatus* genome sequences by constructing a maximum likelihood phylogenetic tree based on a recombination-filtered core genome alignment. Interestingly, the three strains of *L. crispatus* from mother/daughter pair I, UMP1M1, UMP1M2 and UMP1D1, were more similar to each other than to any of the other strains, including others isolated from the female reproductive tract (**Figure 3**).

We also calculated the number of SNPs between our isolates using the recombination-filtered core genome alignment. There were 11 recombination-filtered SNPs between the 2 isolates from the mother (UMP1M1 and UMP1M2) and 25 and 16 recombination-filtered SNPs between the daughter’s isolate (UMP1D1) and the 2 isolates from the mother (UMP1M1 and UMP1M2, respectively).

### 3.5 Estimate of *in vivo* doubling time and mutation rate for vaginal *L. crispatus*

To further investigate the plausibility that the *L. crispatus* strain isolated from daughter I descended from a strain transmitted from her mother at birth, we estimated the doubling time that would allow our isolates to acquire the observed number of SNPs over 20 years. Based on the 25 SNPs between UMP1M1 and UMP1D1, the estimated doubling time for *L. crispatus in vivo* would be 13.2 hours. Based on the 16 SNPs between UMP1M2 and UMP1D1, the estimated doubling time would be 20.6 hours. We also estimated the *in vivo* mutation rate of the core genome of the *L. crispatus* isolates to be 0.4-0.6 mutations per year.

## 4 Discussion

Our study provides preliminary evidence that the vaginal microbiota may be vertically transmitted from mother to daughter at birth *via* vaginal delivery and persists into adolescence. Because the daughters in our study were 15-21 years old, both transmission and persistence were required to observe evidence of vertical transmission. The first piece of evidence supporting vertical transmission is that the vaginal microbiotas of mothers and their adolescent daughters were more similar if their daughter was born by vaginal delivery rather than C-section. The second piece of evidence supporting vertical transmission and persistence is that an important member of the vaginal microbiota, *L. crispatus*, isolated from a vaginally-born, 20-year-old daughter and her mother (pair I) had highly similar genome sequences.

*Lactobacillus* dominance may not be typical in young children but very few vaginal microbiota studies include samples prior to menarche (France et al., 2022a). By ages 10-12, prior to menarche, one study found *Lactobacillus* dominance to be common and the vaginal microbiotas resemble reproductive-stage subjects (Hickey et al., 2015). If *Lactobacillus* persists from birth to adolescence, it may require persistence at low levels for many years. A recent study provides evidence of persistence of several vaginal bacterial strains in reproductive age women for more than a year (France et al., 2022b).

Other studies have compared the vaginal microbiotas of mothers and daughters without analyzing the effect of birth mode (Lee et al., 2013; Hickey et al., 2015; Si et al., 2016; France et al., 2022c). One study found greater similarity between the vaginal microbiotas of mothers and daughters than between unrelated subjects (Lee et al., 2013). This was similar to the trend we observed toward greater community similarity within mother/daughter pairs, regardless of birth mode, than between unrelated subjects in different mother/daughter pairs (**Figure 2A**). Greater similarity between the vaginal microbiota of mothers and daughters was not detected in the other studies (Hickey et al., 2015; Si et al., 2016; France et al., 2022c). If many of the daughters in the other studies were born by C-section then high similarity between mothers and daughters would not be expected. With C- section rates of ~30% in the United States (study site for (Hickey et al., 2015; France et al., 2022c)) and ~36% in South Korea (study site for (Si et al., 2016)) this is a possibility (Caughey et al., 2014; Kim et al., 2017). Additionally, our study focused on adolescent daughters (age 15-21) while the Hickey et al. (2015) study focused on younger daughters and the Si et al. (2016) study focused on older daughters. Reproductive stage has been shown to influence the structure of the vaginal microbiota (Brotman et al., 2018) and we see a similar effect in this study (**Supplemental Figure 2**). Therefore differences in reproductive stage may also contribute to differences in vaginal community composition between mothers and daughters.

Although an overall community similarity was not observed in these studies, specific community members (*Lactobacillus* and *Prevotella*) were identified as most heritable in one study (Si et al., 2016).

Based on the number of SNPs observed between the mother and daughter *L. crispatus* isolates and published mutations rates for *L. casei* Zhang (Wang et al., 2017), we estimated that *L. crispatus* would have an *in vivo* doubling time of 13.2-20.6 hours, depending on the specific isolates compared. The doubling time estimates of 13.2 hours and 20.6 hours for *L. crispatus in vivo* are within the range estimated for other bacteria in their natural environments, including *Escherichia coli* (15 hours) and *Salmonella enterica* (25 hours) (Gibson et al., 2018). These doubling times are faster than the 4.1-5.6 days doubling times measured for *L. casei* Shirota in mouse intestines, where its growth rate was insufficient to maintain colonization (Lee et al., 2004). Although the actual growth and mutation rates of *L. crispatus* in the human vagina have not been measured, we estimated reasonable *in vivo* doubling times for vaginal *L. crispatus* based on the observed number of SNPs between *L. crispatus* isolates from mother/daughter pair I, the age of daughter I and *L. casei* Zhang mutation rates. Considering the uncertainty in the estimates, transmission of *L. crispatus* from mother to daughter at birth followed by the accumulation of independent mutations during 20 years of persistence in the mother and daughter is a plausible explanation for the observed recombination-filtered SNPs. Future studies comparing genomes of *L. crispatus* isolates from more mother/daughter pairs with a variety of daughter ages are needed. Future metagenomic studies could also be used to infer *in vivo* growth rates (Korem et al., 2015).

The 2 *L. crispatus* isolates from the mother had highly similar genomes, differing by only 11 recombination-filtered SNPs. A previous study also observed high similarity between the genomes of multiple vaginal *L. crispatus* isolates from one individual, noting that they were indistinguishable (Abdelmaksoud et al., 2016). Future investigations of *L. crispatus* genomic variation within an individual may yield further insight on colonization and dynamics of the vaginal microbiota.

Consistent with a previous study, *L. crispatus* isolates from the human vagina were phylogenetically intermixed with isolates from the human urinary tract, including highly similar vaginal (ERS1867668 (SAMEA104208650)) and bladder (ERS1867667 (SAMEA104208649)) isolates from the same subject (**Figure 3**) (Thomas-White et al., 2018).

The health implications of vertical transmission of the vaginal microbiota are unknown and were not addressed in this study. However, if vertical transmission is an important factor in determining the composition of the vaginal microbiota there may be important consequences. Vertical transmission of the vaginal microbiota may be one mechanism for maintaining human microbiota over generations *via* a consistent and specific seeding of the newborn microbiota. Delivery mode is an important factor in determining the early composition of the gut microbiota (Rutayisire et al., 2016; Wampach et al., 2018) and is a risk factor for development of immune-related disorders later in life (Tamburini et al., 2016). This suggests an important role for the mother’s vaginal microbiota in seeding the infant and setting the stage for development of the gut microbiota. Therefore, maintenance of the vaginal microbiota between generations may be critical for gut microbiota development in each generation.

Additionally, the vaginal microbiota plays an important if not well understood role in reproductive health, with associations between vaginal microbiota composition and infection susceptibility, bacterial vaginosis (BV) and preterm birth (Callahan et al., 2017; Klatt et al., 2017; McClelland et al., 2018; Bayigga et al., 2019; Tamarelle et al., 2019; Sun et al., 2022). Evidence from this study suggests that transmission of microbes from mother to daughter at birth may influence the composition of the daughter’s microbiota later in life and may contribute to the maintenance of specific members of the human vaginal microbiota over generations.

This study provides evidence consistent with vertical transmission contributing to the vaginal microbiota. As this was a preliminary study, there are some key limitations. This was a small study with only 13 mother/daughter pairs (92% white) and 3/13 daughters born by C-section. Mothers with daughters born by C-section were on average older than mothers with daughters born by vaginal delivery (**Table 1** and **Supplemental Table 2**) and two of the three mothers with daughters born by C-section were post-menopausal which could also contribute to a greater difference in mother/daughter vaginal microbiotas (Brotman et al., 2018). Beyond birth mode and reproductive status, other factors including genetics and shared environment could contribute to mother/daughter vaginal microbiota similarity. Of the eleven pairs asked about cohabitation, only one pair (IV) reported that they didn’t currently live together full or part-time (**Supplemental Table 2**). Therefore, the influence of cohabitation on vaginal microbiota similarity could not be addressed in our study. Genomic analysis of isolates was limited to one member of the vaginal microbiota from 1 mother/daughter pair. Despite these limitations, we feel that these results are intriguing and provide impetus for future studies in larger populations, including more racially diverse subjects, more daughters born by C-section and analysis of more isolate genome sequences or metagenomes to validate these findings.

## Data availability statement

The datasets presented in this study can be found in online repositories. The names of the repository/repositories and accession number(s) can be found below: https://www.ncbi.nlm.nih.gov/bioproject/PRJNA547595; https://www.ncbi.nlm.nih.gov/bioproject/PRJNA547620; https://github.com/cbassis/MotherDaughter_Vaginal_Microbiota.study.

## Ethics statement

The studies involving human participants were reviewed and approved by University of Michigan IRB. Written informed consent to participate in this study was provided by the participants’ legal guardian/next of kin.

## Author contributions

CB was involved in study design and planning, data analysis, figure preparation and manuscript writing. KB was involved in subject recruitment, sample processing, isolation of *L. crispatus* genomic DNA for sequencing, data analysis and manuscript editing. DS was involved in subject recruitment, sample processing and manuscript editing. KS was involved in genomic data analysis, interpretation of genomic data, phylogenetic tree construction and manuscript editing. AP was involved in genomic data analysis and manuscript editing. ES was involved in genomic data analysis and interpretation and manuscript review. VA was involved in subject recruitment and planning. EQ was involved in study design and planning, subject recruitment and manuscript editing. VY was involved in study design and manuscript editing. JB was involved with study design and planning, subject recruitment and manuscript editing. All authors contributed to the article and approved the submitted version.

## Supplementary material

Supplementary Figure 1 Principal coordinates analysis (PCoA) of vaginal microbiota from 13 mother/daughter pairs. The θYC distances between 101 vaginal microbiota samples are represented by PCoA. Samples from daughters are represented by triangles and samples from mothers by circles. Each mother/daughter pair is represented by a unique color. Biplot arrows represent the 3 OTUs most correlated with position on the PCoA plot.

Supplementary Figure 2 Mean θYC distances between subjects within a mother/daughter pair by the mother’s reproductive stage. Wilcoxon (Mann-Whitney) test p-value is shown. In the box and whiskers plots, the median thetaYC distance is indicated by a line, values within the first to the third quartiles are inside the box and the whiskers extend to the smallest and largest values within 1.5x the interquartile range.
