# Supplementary figures and images for: Vaginal microbiota of adolescents and their mothers: A preliminary study of vertical transmission and persistence
Source: Front Microbiomes. 2023 Mar 24;2:1129394. doi: 10.3389/frmbi.2023.1129394 (PMC12993637; doi:10.3389/frmbi.2023.1129394)

Supplemental Figure 1

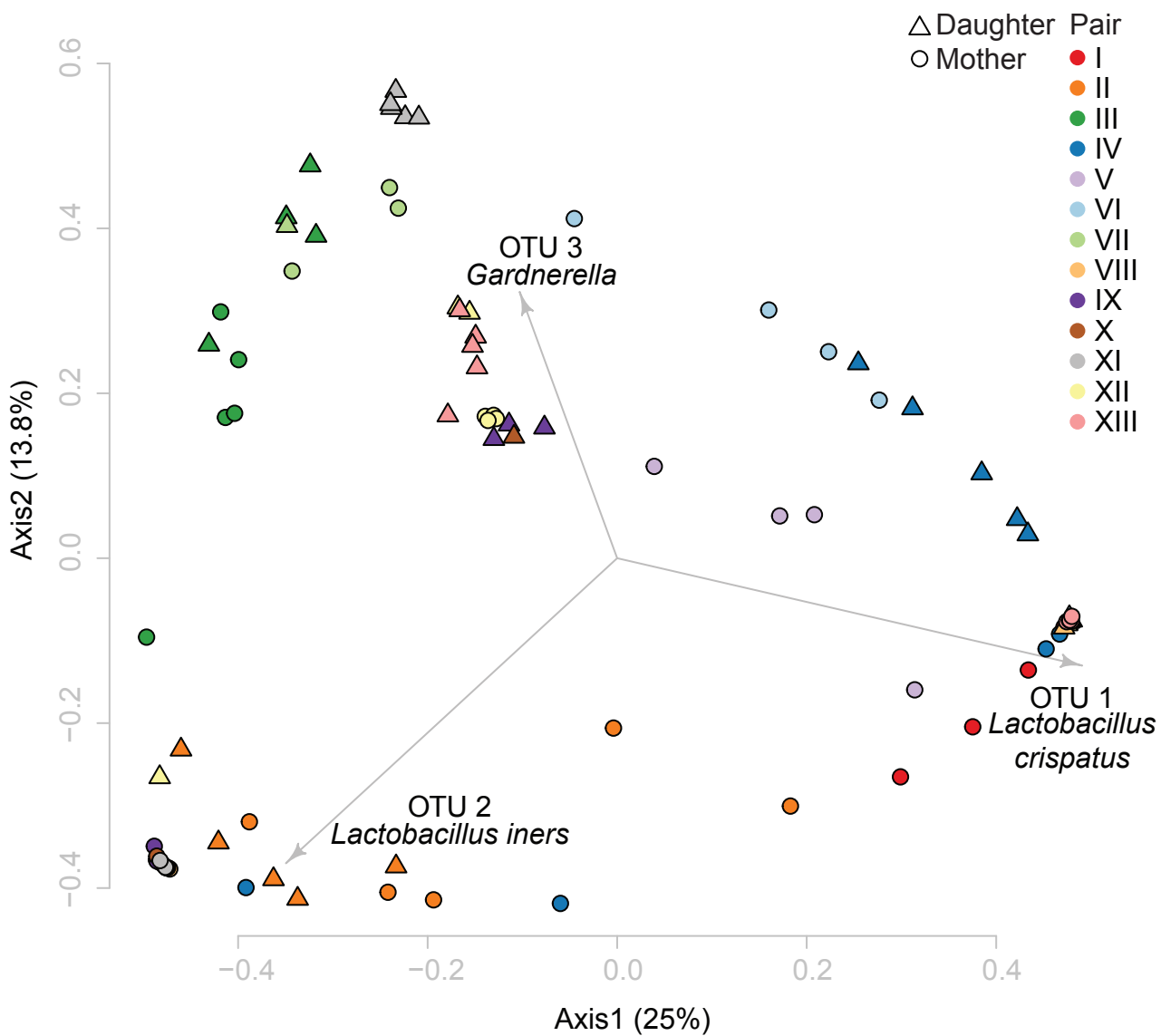

Supplement: Supplementary Figure 1 — Principal coordinates analysis (PCoA) of vaginal microbiota from 13 mother/daughter pairs. The θYC distances between 101 vaginal microbiota samples are represented by PCoA. Samples from daughters are represented by triangles and samples from mothers by circles. Each mother/daughter pair is represented by a unique color. Biplot arrows represent the 3 OTUs most correlated with position on the PCoA plot. [file DataSheet_1.zip › Supplemental Figure 1.PDF]

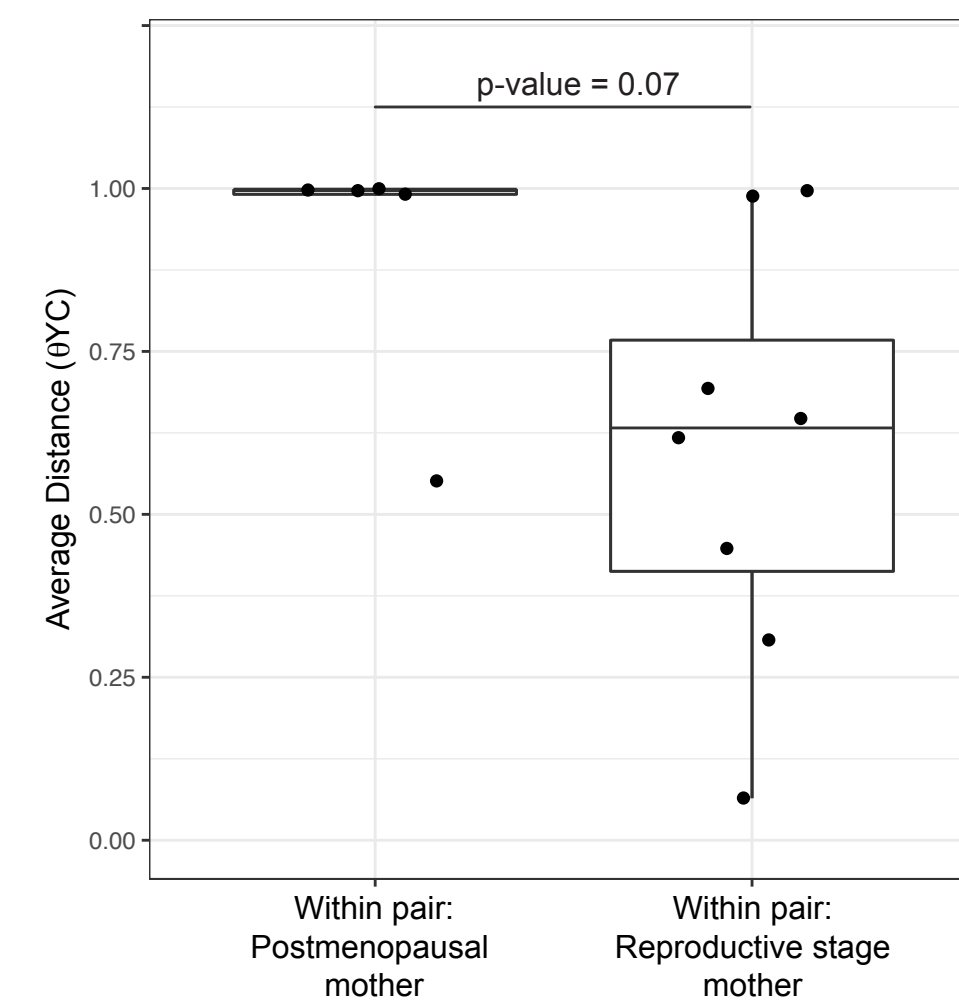

Supplement: Supplementary Figure 1 — Principal coordinates analysis (PCoA) of vaginal microbiota from 13 mother/daughter pairs. The θYC distances between 101 vaginal microbiota samples are represented by PCoA. Samples from daughters are represented by triangles and samples from mothers by circles. Each mother/daughter pair is represented by a unique color. Biplot arrows represent the 3 OTUs most correlated with position on the PCoA plot. [file DataSheet_1.zip › Supplemental Figure 2.PDF]
